# Enhancing Global Cognition and Executive Functions Through Water-Based Exercise in Mild Cognitive Impairment: A Randomized Controlled Trial

**DOI:** 10.3390/life15030420

**Published:** 2025-03-07

**Authors:** Sutaya Meekum, Kornanong Yuenyongchaiwat, Nongnuch Luangpon, Piyapa Keawutan, Patcharee Kooncumchoo

**Affiliations:** 1 Department of Physical Therapy, Faculty of Allied Health Sciences, Thammasat University, Pathum Thani 12120, Thailand; sutaya.mee@allied.tu.ac.th (S.M.); kornanong.y@allied.tu.ac.th (K.Y.); piyapa.k@allied.tu.ac.th (P.K.); 2 Thammasat University Research Unit for Physical Therapy in Respiratory and Cardiovascular Systems, Thammasat University, Pathum Thani 12120, Thailand; 3 Department of Physical Therapy, Faculty of Allied Health Sciences, Burapha University, Chon Buri 20131, Thailand; nongnuchl@go.buu.ac.th; 4 Center of Excellence in Creative Engineering Design and Development, Thammasat University, Pathum Thani 12120, Thailand

**Keywords:** mild cognitive impairment, water-based exercise, older adults

## Abstract

Mild cognitive impairment (MCI) is a slight cognitive decline with the ability to perform normal activities in daily life and an increased risk of dementia. Land-based exercise enhances cognitive abilities, but combining cognitive and physical interventions offers greater benefits in MCI. Water-based exercise is a low-impact activity that minimizes joint strain and reduces injury risk. This study investigated the effects of water-based exercise with or without cognitive training on cognition in older adults with MCI. Thirty-seven participants aged 65 years or older with MCI were randomly assigned to two groups: water-based exercise (W; n = 18) and water-based exercise combined with cognitive training (W-COG; n = 19). Both groups performed 60 min water-based aerobic sessions 3 days per week for 12 weeks. Cognitive assessments were conducted at baseline and after 12 weeks. Post-intervention, 77.77% of the W group and 89% of the W-COG group had improved their cognitive ability. Both groups showed significant improvements in their global cognition (*p* < 0.001, *p* < 0.001) and visuospatial ability (*p* < 0.01 for W, *p* < 0.05 for W-COG), respectively. In addition, cognitive flexibility and shifting abilities improved only in the W-COG group (*p* < 0.05). These findings suggest that water-based exercise combined with cognitive training enhances cognitive functions more effectively than exercise alone in older adults with MCI.

## 1. Introduction

Mild cognitive impairment (MCI) is a transitional stage between normal age-related cognitive decline and the preservation of independence in the activities of daily living [1]. Mild cognitive impairment affects approximately 8.4% of adults aged 65 to 69, 10.1% of those aged 70 to 74, 14.8% of those aged 75 to 79, and 25.2% of those aged 80 to 84, according to the American Academy of Neurology [2]. Memory, attention, executive function (EF), language, and visuospatial skills are the most affected domains in older adults with MCI [3]. The mean age of older Thai people with MCI was 68.3 ± 6.82 years, and most had an education of ≤4 years. Considering the cognitive subdomains assessed by the Thai version of the MoCA-B, executive function, alternating attention, and delayed recall were the most common cognitive impairments [4]. In addition, older adults with mild cognitive impairment (MCI) tend to progress to dementia at a rate of up to 7.1% per year. People with MCI and mild dementia have a significantly increased risk of worsening over the next several years [5]. There are several risk factors for the progression from MCI to dementia, including age [6], vascular diseases [7], lower levels of formal education and intellectual activity [8], depression [9], and sleep quality [10].

Previous studies have shown that physical exercise had a positive impact on cognitive function in healthy older adults and those with cognitive impairment, but not in individuals with MCI [11,12,13]. There are many types of physical exercise that can promote physical and cognitive function, such as resistance exercise, aerobic exercise, and multimodal exercise [14,15]. Aerobic exercise has moderate effects on improving global cognition, logical memory, inhibitory control, and divided attention in older adults with MCI [13]. A moderate or higher intensity of aerobic exercise (with a total training time > 24 h) has a more pronounced effect on global cognition [15]. Furthermore, land-based exercise combined with cognitive training tends to be more effective for promoting cognition and functional activities than exercise alone in older adults with MCI [16].

The results for multimodal exercise in older adults with MCI have suggested the benefit of exercise for cognitive performance. The combination of aerobic exercise, muscle strength training, and balance exercise for 90 min and 25 min of dual-task exercise for one session/week for 40 weeks showed significant improvements in physical performance, daily physical activity, and cognitive abilities and logical memory in the combined activity group [17]. Moreover, the combination of physical exercise and brain training or cognitive training for 25–60 min showed a superior effect for older adults with MCI than exercise alone.

Cognitive training combined with physical training showed a significantly greater improvement in transfer and basic cognitive functions, memory, and attention. The results showed a lower number of errors in all tests and greater visual–motor coordination compared with physical training alone [18]. The combined cognitive games and aerobic exercise group showed more improvement in walking tests and executive function than those who received cognitive training alone [19]. The dual-task memory exercise and aerobic exercise showed an improvement in functionality during transfers (turning and sitting down), but not in cognition [20].

Water-based exercise is fun, enjoyable, and serves as an alternative form of exercise that is suitable for applying to older people with MCI. The benefit of the exercise comes from the buoyant force of the water that supports the body, which makes movement easier, reduces the impact on the joints, and lowers the risk of injury from exercise. During exercises in water, the subject is required to exert the maximum strength of both their upper and lower body muscles against the water pressure in all directions. This allows for efficient energy metabolism with a low risk of injury, as well as reducing the chance of falling due to the support from the water [21]. According to physics calculations, the body weight is reduced by approximately 50% when water is at waist level and reduced to 30% of the original body weight when the water level reaches the chest. Walking exercise in water at chest level also increases respiratory muscle strength. The respiratory muscles were found to be more active than the respiratory muscles of those who walked on land because of the hydrostatic pressure limiting the expansion of the thorax while breathing. As a result, it was necessary to exert more force than usual to breathe and increase the expansion of the chest, leading to the respiratory muscles working more than in a usual situation [22]. Few studies have examined the effectiveness of water-based exercise in improving cognitive performance in older adults with MCI. The aims of this study were to evaluate the effectiveness of water-based exercise alone or combined with cognitive training in improving cognitive performance in older adults with MCI.

## 2. Materials and Methods

**Figure 1:**
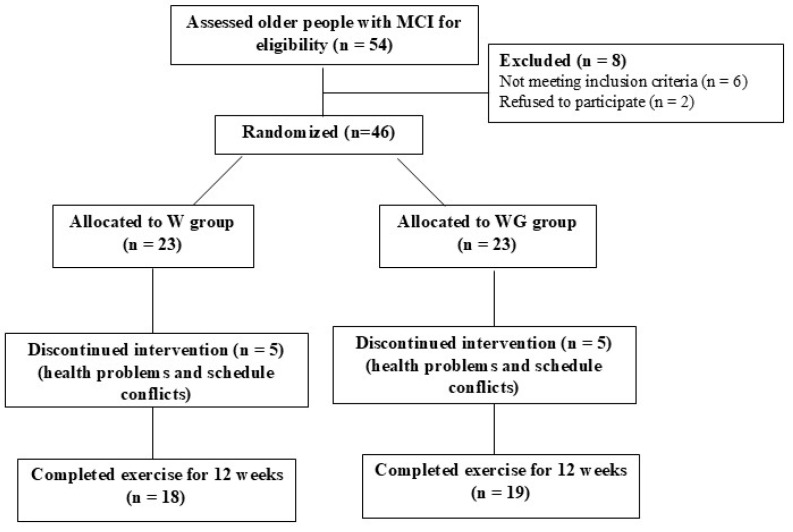
A CONSORT diagram showing the flow of participants in the study.

This study used a single-blind, clustered, randomized, controlled trial design. A computer program was used to randomly assign participants to each group. Thirty-seven participants with mild cognitive impairment, aged 65 years old or older, were recruited into the study. They were screened by using the MCI diagnostic criteria, had MoCA scores between 17 and 24 points, a Lawton Instrumental Activities of Daily Living Scale (LIADL) score of ≥8 points, and had no evidence of significant impairment in social or occupational functioning [23]. Participants were randomly assigned to 2 groups (Figure 1): a water-based exercise group (W group) and a water-based exercise combined with cognitive training group (W-COG group). The intervention involved small group exercises (4–5 participants). Participants were excluded from the outcome analysis if they missed consecutive exercise sessions for a period of 3 days or more or if they missed a total of 8 sessions.

The participants in the water-based exercise group performed circuit aerobic exercise in water (12 exercises, 10 repetitions per exercise, with a progression to 20 repetitions per exercise after 4 weeks and 30 repetitions per exercise after 8 weeks). The intensity of aerobic exercise was set at a moderate level (50–70% of the Maximum Heart Rate), monitored using a smartwatch. Heart rates were monitored and recorded individually during each session. Each session lasted for 60 min (40–45 min of exercise, with 15–20 min for a warm-up and cool-down), 3 days per week for 12 weeks. Even though the number of repetitions increased after 4 and 8 weeks, the total exercise time remained within 60 min for the entire 12 weeks, as the participants’ agility improved, allowing them to move quickly and increase their speed [24].

The participants in the water-based exercise combined with cognitive training group received cognitive training by memorizing an exercise protocol displayed on a TV, which was represented by numbers and colors that correlated with the exercise, simultaneously while exercising in the water. The same numbers and colors that correlated with the exercises were used in the same way for 12 weeks. The sequence of circuit aerobic exercises was the same as the exercise in the water-based exercise group. Participants in the water-based exercise combined with cognitive training group received this protocol of 60 min per session 3 days per week for 12 weeks. The protocol aimed to minimize the information given and train participants to recall it on their own after exercise for 4 weeks. The order of the 12 exercises was randomized during weeks 7–12. However, participants were still able to receive information and feedback from their group. At the beginning, the correct exercise activity and feedback were provided to participants immediately if an error was found during exercise. The information and feedback from the TV and trainers decreased after 4 weeks; only numbers or colors were displayed on the TV, and participants were required to remember and recall the number and color that correlated with each exercise. All participants needed to remember and recall exercises on their own after hearing the number or color during each exercise after 8 weeks.

Cognitive performance was evaluated based on three main components: global cognition, executive function, and working memory, both before and after the intervention. Global cognition was assessed using the Montreal Cognitive Assessment (MoCA)—Thai version; executive function (visuospatial ability) was assessed using Trail-Making Test A (TMT-A); and executive function (visuospatial ability and cognitive flexibility) was assessed using Trail-Making Test B (TMT-B). Additionally, Trail-Making Test B-A measured the shifting ability. Executive function (cognitive flexibility, selective attention, visuospatial ability, and inhibitory control) was assessed using the Stroop Color and Word Test (SCWT), while working memory was evaluated through the use of the Digit Span Forward and Backward Tests (DSFT and DSBT).

An independent Student’s *t*-test and chi-square test were used to compare the differences in the baseline general characteristics between the two groups, where appropriate. A two-way mixed analysis of variance was used to analyze the variables both within and between groups. The statistical significance level was set as 0.05. The observed power for the MoCA (within) was found to be 1.00, for the MoCA (between) was 0.322, for the TMT-A was 0.9 for the W group or 0.573 for the W-COG group, and for the TMT-A (between) was 0.089.

## 3. Results

Forty-six older adults with mild cognitive impairment participated in the study. They were divided into two groups through simple random sampling by a computer program: the water-based exercise group (W group; n = 23) and the water-based exercise combined with cognitive training group (W-COG group; n = 23). During the 12-week intervention period, five participants dropped out of the W group (21.7%) and four participants out of the W-COG group (17.4%) due to health problems or schedule conflicts. The adherence rates for participants who completed the 12 weeks were 78.3% (n = 18) in the W group and 82.6% (n = 19) in the W-COG group (Figure 1).

### 3.1. General Characteristics of Participants

The average ages of participants in the W group and the W-COG group were 68.3 ± 3.3 and 69.6 ± 3.8 years old, respectively. Most participants in this study were female and had an education level equal to primary school or less than 6 years. Both groups had similar numbers of underlying diseases and had an equal number of exercise or non-exercise behaviors (Table 1). There were no significant differences in the general characteristics at baseline between the two groups.

### 3.2. Cognitive Performance

An improvement in cognitive performance regarding global cognition and executive function was found in both groups after 12 weeks of intervention. The W group improved in terms of global cognition and executive function (visuospatial ability). Improvements in global cognition and executive functions (visuospatial ability, cognitive flexibility, and shifting ability) were found in the W-COG group. A two-way mixed model ANOVA was performed to evaluate the impact of the exercise program and duration on three domains of cognitive performance. In this study, all variables followed a normal distribution. Outlier data were excluded because their test scores differed from those of the rest of the group by more than two standard deviations. This was performed to accurately assess the effectiveness of the exercise programs in both groups. Outlier data were identified for the MoCA variable (W group; one participant), the SCWT variable (W group: two participants; W-COG group: one participant), the TMT-A variable (W-COG group; one participant), and the DSBT variable (W-COG group; one participant).

An improvement in global cognition was observed after 12 weeks (Table 2). Significant changes in the MoCA score were found in the W and the W-COG groups. The MoCA score increased from 21.88 ± 1.79 to 26.25 ± 2.46 in the W group (*p* < 0.001) and from 22.37 ± 1.16 to 27.32 ± 2.08 in the W-COG group (*p* < 0.001). However, there were no significant changes in the MoCA score between the two groups.

In addition, when observing the improvement in normal cognition based on the MoCA scores (a MoCA score of ≥25 points indicates normal global cognition), the study found that there were no participants with normal cognition at baseline. However, after 12 weeks of exercise, the MoCA scores improved in both groups. Most participants showed an improvement in normal cognition: 77.77% (14 people) in the W group and 89% (17 people) in the W-COG group. These results suggested the effectiveness of water-based exercise alone or in combination with cognitive training for improving cognition after 12 weeks of exercise in older adults with MCI.

**Figure 2:**
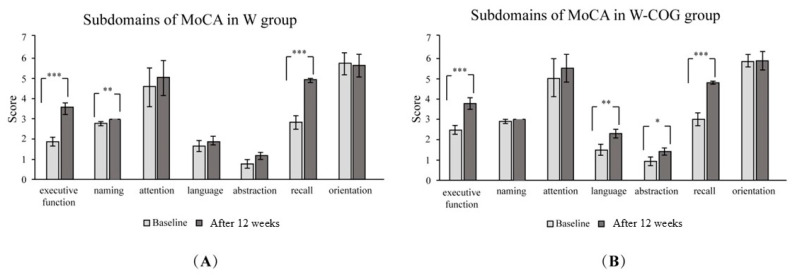
Subdomains of MoCA scores at baseline and after exercise for 12 weeks: (**A**) W group; (**B**) W-COG group. * indicates *p* > 0.05, ** indicates *p* > 0.01, *** indicates *p* > 0.001.

When considering the subdomains of the MoCA assessment, there was an increase in the subdomains for executive function (*p* < 0.001), naming (*p* < 0.005), and recall memory (*p* < 0.001) in the W group (Figure 2A). In the W-COG group (Figure 2B), there was an increase in the subdomains for executive function (*p* < 0.001), language (*p* < 0.005), abstraction (*p* < 0.05), and recall memory (*p* < 0.001).

Changes in executive function were found in both groups after 12 weeks of exercise, specifically in terms of their visuospatial ability, measured by the TMT-A. Significant changes were found in the W group and the W-COG group. The time to complete the TMT-A decreased from 65.72 ± 23.25 to 51.11 ± 14.32 s in the W group (*p* < 0.01) and from 57.44 ± 20.07 to 47.78 ± 19.05 s in the W-COG group (*p* < 0.05). There were no significant differences between the two groups.

Moreover, the visuospatial ability and cognitive flexibility domains in executive function were assessed using the TMT-B. There were no differences in the TMT-B times between the two groups. However, significant changes in the visuospatial ability and cognitive flexibility domains were observed between the groups after controlling for their age *(p* < 0.05) or educational level *(p* < 0.05).

The shifting ability was evaluated using the TMT B-A. The findings were similar to those for the TMT-B. There were no differences in the TMT-B times between the two groups after 12 weeks. However, after controlling for age and educational level, a significant decrease was found in the W-COG group compared to the W group *(p* < 0.05). No significant differences in the shifting ability were found in either the W group or the W-COG group after 12 weeks of exercise.

Cognitive flexibility, selective attention, visuospatial abilities, and inhibitory control were assessed using the SCWT time. There were no significant differences either between the two groups or within the groups. Although there were no significant differences after 12 weeks of exercise, the time to complete the SCWT in the W group showed a tendency to increase from 62.81 ± 21.42 to 77.5 ± 26.58 s, while in the W-COG group it showed a tendency to decrease from 65.61 ± 34.64 to 55.44 ± 23.52 s. An increase in time on the SCWT indicates greater difficulty in processing the task, while a decrease in time indicates an improvement in focus and the ability to process tasks with conflicting information.

Working memory was assessed using the Digit Span Forward Test (DSFT) and the Digit Span Backward Test (DSBT). No significant changes in working memory were found in the DSFT or DSBT after 12 weeks of exercise, either between the two groups or within the groups.

## 4. Discussion

An improvement in global cognition and executive function (visuospatial ability) was found in both groups. However, an improvement in executive function (cognitive flexibility and shifting ability) was observed only in the W-COG group. The improvement in global cognition and executive function in both groups may have been related to a moderate intensity of aerobic exercise over a period of 12 weeks. The increase in the heart rate during exercise leads to increased cerebral blood flow, oxygen levels, and neurotransmitter transmission in the brain [25]. The combination of water-based aerobic exercise and resistance training in this study may have been more beneficial for improving cognitive abilities. A recent meta-analysis suggested that a combination of aerobic exercise and resistance training is the most effective method for improving cognition in older people with MCI, while resistance exercise alone is the most effective for dementia [26].

Exercising in water promotes greater and more intense movement for older adults due to specific properties such as buoyancy and water viscosity, which promote greater assistance/resistance during movement. These encourage participants to use their body and move with wider ranges while exercising in water [27]. Moreover, the group water-based exercise program, either alone or combined with cognitive training, may have provided a fun and enjoyable environment and motivated the elderly to exercise at a moderate intensity throughout the training period. Both interventions may have provided an enriched environment in which individuals were involved in mentally stimulating tasks while being physically active [28]. A moderate or higher intensity of aerobic exercise had a greater effect on global cognition [15]. The combined cognitive and physical exercise programs showed an improvement in global cognition in older adults with MCI, especially of the amnestic group.

Previous studies showed that an increase in functional fitness and cognitive functions, including global cognition, were found with moderate-intensity aquatic exercise for 30 min per day (28 weeks) in non-institutionalized elderly. A total of 16 weeks of water-based exercise increased brain-derived neurotrophic factor (BDNF), related to increased cognitive performance in healthy elderly women [29]. BDNF plays a crucial role in physical exercise for maintaining cognitive function and enhancing neuronal plasticity [30], including nourishing neurogenesis and improving neuronal survival [31]. Moreover, an increase in the hippocampal volume was observed in elderly women after resistance training for 50–80 min a day, three times per week, over a total duration of 24 weeks [32] or in Parkinson’s disease after 6-week exergaming training combined with moderate-intensity aerobic exercise [33].

The improvement in executive function regarding visuospatial abilities (TMT-A) in both groups in this study resembles the findings of a previous study involving land-based or aquatic-based exercise for 6 months, followed by subsequent cognitive training over 4 weeks, in older adults with MCI [28]. An improvement in visuospatial abilities and cognitive flexibility (TMT-B) and shifting abilities (TMT B-A) was observed only in the W-COG group (Table 2). The TMT-B is known to be more complex and challenging to perform than the TMT-A. These results suggest that exercise combined with cognitive training was more effective than exercise alone for improving visuospatial abilities, cognitive flexibility, and shifting abilities in older adults with MCI. However, the lack of significant differences after 12 weeks of exercise in both groups may be due to difficulties in test performance related to participants’ low educational background and age, which may have contributed to their challenges in completing the test [34].

The design of the cognitive training program in this study was specific and challenging in terms of executive function. The cognitive training program, which randomized the sequence of exercises during weeks 7–12, required participants to focus on the trainer’s instructions, thereby challenging their concentration more than the W group, which followed the same sequence throughout the intervention. Most participants were able to remember and independently follow the exercise, but some participants only repeated and followed the movements of their friends. This may have influenced the cognitive results of the study, as some participants were unable to recall and perform exercise on their own. Randomized tasks may require participants to continually adjust their actions, thereby engaging their cognitive flexibility and shifting ability more than repetitive, predictable exercises [35]. The training program adhered to the structure of a previously developed “comprehensive extended” training regimen. This encompassed additional non-mnemonic “pretraining”, which involved visualization methods, alongside mnemonic training. The mnemonic training incorporated list-learning tasks and name–face recall [36].

The moderate–high-intensity aquatic-based exercise plus cognitive training improved participants’ executive function, processing speed, language, and visuospatial ability. These cognitive changes may have resulted from increased cerebral blood flow during partial body immersion in water [37], as well as from stimulating various aspects of brain function such as postural control, mechanoreceptors, and parasympathetic drive and leading to neurogenesis and synaptic plasticity changes [38]. Exercising for 30–60 min per session continuously over 12 weeks led to an increase in executive function abilities due to the activation of brain networks and the synthesis of neurotransmitters in healthy individuals [39]. The incorporation of various physical exercises such as aerobics, muscle strengthening, and gross motor activities may have contributed to the improvement in executive function [40].

However, this study found a significant difference in the cognitive flexibility and shifting ability in the W-COG group compared to those in the W group, but not in other domains of cognition. This may be due to both groups receiving the same moderate intensity of water-based aerobic exercise for 12 weeks. Therefore, those participating in the combined cognitive training program likely experienced increased cognitive demand, potentially enhancing neurogenesis and synaptic plasticity [38].

A 12-week dual-task training program (combining balance and strengthening with cognitive training) in older participants did not yield significant improvements in their executive function and walking ability when compared to a group receiving only balance and strengthening exercises. The participants were already in good health and physically active; the training did not yield a positive impact on their physical performance [41].

A combined cognitive games and aerobic exercise group showed more improvement in physical performance (walking test) than those engaged in physical training alone [19] or a greater improvement in cognitive abilities [17]. In contrast to this study, their cognitive abilities showed less improvement with combined cognitive training compared to exercise alone. These differences may be due to the variations in the combined cognitive training protocol, such as setting up a small group exercise, training recall simultaneously with exercise, and being less targeted across multiple cognitive domains. The combined cognitive training in previous studies was more intense and targeted a wider range of cognitive domains and was delivered in an individualized training session, which may have enhanced participant engagement and focus on the cognitive training program. These factors could potentially have affected differences in the improvement in cognitive abilities.

The lack of changes in working memory may have been due to the protocol of cognitive training and the cognitive performance of the participants in this study. The cognitive training program in the study primarily focused on recall memory through remembering exercise movements. Moreover, this study did not include specific variables to assess recall memory, which might explain the lack of observed differences. Moreover, the normal scores for the Digit Span Forward and Backward Tests vary based on many factors such as participants’ age, education level, and cognitive ability. For future research, the enhancement of working memory should be explored by adopting a more specific protocol of cognitive training focused on working memory, including controlling other confounding factors such as participants’ age, educational level, and cognitive ability and the frequency and duration of the exercise.

In addition, the participants in this study had normal working memory based on scores from the DSFT (seven digits) and DSBT (three digits), with a low educational background and MCI. The average score for the Digit Span Forward Test is typically five digits, while the average score for the Digit Span Backward Test is typically three digits for older adults [42]. Therefore, these factors may have influenced the improvement in working memory in both groups.

A small sample size may be a limitation for generalizing the results to the entire population. Many participants in this study had a low educational background, were female, and were older. Therefore, these factors may have influenced the difficulty of test performance and should be controlled. Moreover, this study was unable to monitor participants’ real-time heart rates during exercise. The target heart rate was determined using the average heart rate after the exercise sessions to indicate the intensity of exercise. Furthermore, both groups received the same moderate-intensity aerobic exercise program, so differences between the two groups could not be found. For further studies including larger sample sizes, the benefits of water-based exercise should be studied in other older populations, such as those with sarcopenia, a risk of falls, or frailty. Additionally, the exercise duration and follow-up periods should be extended to assess the long-term sustainability of cognitive benefits from exercise.

Clinical Implication: Water-based exercise interventions present a promising nonpharmacological strategy for preventing cognitive decline and improving cognition in older adults with MCI. Exercise interventions, either alone or combined with cognitive training, have the potential to delay cognitive decline, enhance global cognition, and improve executive function. Small group exercise in water, as a fun, relaxing environment with feedback from friends, could be one strategy that helps older adults engage in exercise for longer periods. The interventions may serve as clinical strategies for healthcare professionals specializing in geriatric care, particularly in the context of cognitive function.

## 5. Conclusions

Water-based exercise alone or combined with cognitive training has the potential to enhance cognitive function and improve cognition in older adults with MCI. Global cognition and executive function (visuospatial ability) improved in both programs. Moreover, water-based exercise combined with cognitive training may offer additional benefits for cognitive flexibility and shifting abilities in older adults with MCI.

## Figures and Tables

**Table 1:** General characteristics of participants in the water-based exercise (W) group and the water-based exercise combined with cognitive training (W-COG) group.

Characteristics (Mean ± SD)	W Group (n = 18)	W-COG Group (n = 19)	*t* (Test)/x^2^	*p*-Value
Age, yrs.	68.39 ± 3.35	69.68 ± 3.85	−1.09	0.283
Weight, kg	61.33 ± 8.76	56.36 ± 8.79	1.72	0.094
Height, cm	156.89 ± 5.94	155.68 ± 6.15	0.61	0.549
Body mass index, kg/m^2^	24.60 ± 4.31	23.62 ± 2.70	0.82	0.4120
% skeletal mass	23.02 ± 2.91	22.81 ± 2.20	0.26	0.799
% fat	35.29 ± 5.75	35.60 ± 3.78	−0.19	0.849
MoCA scores	21.61 ± 2.09	22.37 ± 1.16	−1.35	0.188
Resting HR, bpm	76.94 ± 14.89	77.11 ± 9.79	−1.01	0.320
Gender (n)			0.424 ^#^	0.515
Female:male	16:2	18:1		
Education background, n (%)			1.53 ^#^	0.465
- Primary school	13 (72.2%)	10 (52.6%)		
- Secondary school	3 (16.7%)	5 (26.3%)		
- University	2 (11.1%)	4 (21.1%)		
Underlying diseases (n)			3.695 ^#^	0.296
0:1:2: ≥ 3	3:5:5:5	7:7:3:2		
Exercise behavior, n (%)			0.232 ^#^	0.630
<3 days/week	9 (50%)	8 (42.1%)		
≥3 days/week	9 (50%)	11 (57.9%)		

Note: ^#^ analysis by chi-square test; MoCA: Montreal Cognitive Assessment; resting HR: resting heart rate.

**Table 2:** Cognitive performance after exercise for 12 weeks in water-based exercise (W) group and water-based exercise combined with cognitive training (W-COG) group.

Cognitive Tests	Water-Based Exercise (n = 18) Mean ± SD			Significant Within W Gr.	Water-Based Exercise Combined with Cognitive Training (n = 19) Mean ± SD			Significant Within W-COG Gr.	Significant Between W and W-COG Grs.
	Baseline	12 Weeks	Mean Dif.		Baseline	12 Weeks	Mean Dif.		
Montreal Cognitive Assessment (MoCA; score)	21.88 ± 1.79 (n = 17)	26.25 ± 2.46 *** (n = 17)	4.37 ± 0.67	*F* (1, 35) = 74.544, ***p* < 0.001**, ηp^2^ = 0.682	22.37 ± 1.16	27.32 ± 2.08 ***	4.95 ± 0.92	*F* (1, 35) = 92.803, ***p* < 0.001**, ηp^2^ = 0.726	*F* (1, 35) = 2.365, *p =* 0.133, ηp^2^ = 0.063
Digit Span Forward Test (DSFT; digit)	7.44 ± 1.04	7.78 ± 0.87	0.34 ± 0.17	*F* (1, 35) = 1.646, *p* = 0.208, ηp^2^ = 0.045	7.11 ± 1.15	7.26 ± 0.87	0.15 ± 0.28	*F* (1, 35) = 0.390, *p* = 0.536, ηp^2^ = 0.011	*F* (1, 35) = 3.197, *p* = 0.082, ηp^2^ = 0.084
Digit Span Backward Test (DSBT; digit)	3.44 ± 0.78	3.5 ± 0.61	0.06 ± 0.17	*F* (1, 34) = 0.042, *p* = 0.839, ηp^2^ = 0.001	3.72 ± 0.89 (n = 18)	3.72 ± 1.63 (n = 18)	0 ± 0.74	*F* (1, 34) = 0.000, *p* = 1.000, ηp^2^ = 0.000	*F* (1, 34) = 0.279, *p* = 0.601, ηp^2^ = 0.008
Stroop Color and Word Test (SCWT; time)	62.81 ± 21.42 (n = 16)	77.5 ± 26.58 (n = 16)	14.69 ± 5.16	*F* (1, 32) = 1.420, *p* = 0.242, ηp^2^ = 0.042	65.61 ± 34.64 (n = 18)	55.44 ± 23.52 (n = 18)	10.17 ± 11.12	*F* (1, 32) = 2.188, *p* = 0.149, ηp^2^ = 0.064	*F* (1, 32) = 3.492, *p* = 0.071, ηp^2^ = 0.098
Trail-Making Test Part A (TMT-A; time)	65.72 ± 23.25	51.11 ± 14.32 **	14.61 ± 8.93	*F* (1, 34) = 11.130, ***p* = 0.002**, ηp^2^ = 0.247	57.44 ± 20.07 (n = 18)	47.78 ± 19.05 * (n = 18)	9.66 ± 1.02	*F* (1, 34) = 4.872, ***p* = 0.034**, ηp^2^ = 0.125	*F* (1, 34) = 0.352, *p* = 0.557, ηp^2^ = 0.010
Trail-Making Test Part B (TMT-B; time)	186.56 ± 72.40	178.33 ± 69.28	8.23 ± 3.12	*F* (1, 33) = 0.670, *p* = 0. 419, ηp^2^ = 0.020	153.63 ± 68.80	125.63 ± 65.86	28 ± 2.94 ^a^ *^,^^b^ *	*F* (1, 33) = 2.057, *p* = 0. 161, ηp^2^ = 0.059	^a^: *F* (1, 33) = 5.613, ***p* = 0.024**, ηp^2^ = 0.142 ^b^*: F* (1, 33) = 5.813, ***p* = 0.021**, ηp^2^ = 0.146
Trail-Making Test Part B-A (TMT B-A; time)	86.89 ± 49.76	102.61 ± 52.90	6.39 ± 79.50	*F* (1, 33) = 0.010, *p* = 0.921, ηp^2^ = 0.000	127.79 ± 62.21	97.79 ± 59.53	21.16 ± 48.31 ^c^ *	*F* (1, 33) = 1.331, *p* = 0.257, ηp^2^ = 0.039	*F* (1, 33) = 6.775, ***p* = 0.014**, ηp^2^ = 0.170

Note: * indicates *p* < 0.05, ** indicates *p* < 0.01, *** indicates *p* < 0.001, ^a^ indicates significant differences adjusted for age, ^b^ indicates significant differences adjusted for education level, ^c^ indicates significant differences adjusted for age and education level.

## Data Availability

The original contributions presented in this study are included in the article. Further inquiries can be directed to the corresponding author.
